# Prognostic value of circulating neuron-specific enolase combined with erythrocyte and platelet distribution indices in patients with severe traumatic brain injury

**DOI:** 10.5937/jomb0-65680

**Published:** 2026-06-16

**Authors:** Tengyu Li, Dingjun Ma, Guozhen Zhang, Chao Wang, Bin Xu, Weidong Zhu

**Affiliations:** 1 Beijing University of Chinese Medicine, Dongzhimen Hospital, Department of Neurosurgery, Beijing, China

**Keywords:** severe traumatic brain injury, neuron-specific enolase, red cell distribution width, platelet distribution width, prognostic biomarkers, teška traumatska povreda mozga, neuron-specificna enolaza, širina raspodele eritrocita, širina raspodele trombocita, prognosticki biomarkeri

## Abstract

**Background:**

Severe traumatic brain injury (STBI) is associated with high mortality and long-term neurological disability. Early identification of reliable circulating biomarkers may improve prognostic stratification and clinical management. Haematological distribution indices, including red cell distribution width (RDW) and platelet distribution width (PDW), along with neuron-specific enolase (NSE), reflect systemic inflammation, platelet activation, and neuronal injury following traumatic brain injury. However, their combined prognostic value in STBI remains incompletely understood.

**Methods:**

A retrospective study was conducted, including 96 consecutive patients with STBI admitted between March 2020 and March 2023. The GCS score used for rSIG calculation was the first documented admission score before definitive neurosurgical treatment. Treatment was individualised according to injury severity and imaging findings, including conservative management, hematoma evacuation, decompressive craniectomy, ventilatory support, and intensive care when indicated. Peripheral blood levels of RDW, PDW, and serum NSE were measured at admission using automated haematology analysis and enzyme-linked immunosorbent assay. According to the Glasgow Outcome Scale (GOS), evaluated 3 months after injury, patients were classified into a good-prognosis group (n= 46) and a poor-prognosis group (n = 50).

## Abstract (sr)

**Uvod:**

Teška traumatska povreda mozga (TTPM) je povezana sa visokom smrtnošću i dugotrajnim neurološkim invaliditetom. Rano prepoznavanje pouzdanih cirkulišućih biomarkera može unaprediti prognosticku stratifikaciju i klinicko upravljanje pacijentima. Hematološki indeksi raspodele, uključujući širinu raspodele eritrocita (RDW) i širinu raspodele trombocita (PDW), zajedno sa neuron-specificnom enolazom (NSE), odražavaju sistemsku infla-maciju, aktivaciju trombocita i oštećenje neurona nakon traumatske povrede mozga. Međutim, njihov kombinovani prognosticki znacaj kod TTPM još uvek nije u potpunosti razjašnjen.

**Metode:**

Sprovedena je retrospektivna studija koja je obuhvatila 96 uzastopnih pacijenata sa TTPM, primljenih u periodu od marta 2020. do marta 2023. godine. GCS skor korišćen za izračunavanje rSIG indeksa bio je prvi zabeleženi prijemni skor pre definitivnog neurohirurškog lečenja. Terapija je individualno prilagođavana u skladu sa težinom povrede i nalazima sa snimaka, uključujući konzervativno lečenje, evakuaciju hematoma, dekompresivnu kraniektomiju, respiratornu potporu i intenzivno lečenje prema indikacijama. Nivoi RDW i PDW u perifernoj krvi, kao i serumski NSE su određivani pri prijemu pomoću automatizovane hematološke analize i enzimskog imuno-eseja (ELISA). Na osnovu Glasgow Outcome Scale (GOS), tri meseca nakon povrede, pacijenti su podeljeni u grupu sa dobrim ishodom (n = 46) i grupu sa lošim ishodom (n = 50).

**Rezultati:**

Pacijenti sa lošim ishodom su imali značajno više vrednosti RDW, PDW i NSE, kao i niže rSIG vrednosti u poređenju sa pacijentima sa povoljnim ishodom (P&lt;0,05). Multivarijantna logistička regresiona analiza je identifikovala povišene vrednosti RDW, PDW i NSE, kao i snižene vrednosti rSIG, kao nezavisne faktore povezane sa nepovoljnim ishodom kod pacijenata sa TTPM. ROC analiza je pokazala umerenu prediktivnu vrednost za RDW (AUC=0,737), PDW (AUC=0,749), NSE (AUC=0,736) i rSIG (AUC=0,752). Kombinovani model biomarkera pokazao je bolju diskriminativnu sposobnost (AUC=0,793), uz visoku senzitivnost (95,00%), ali ograničenu specifičnost (50,00%).

**Zaključak:**

Cirkulišuća NSE, zajedno sa hematološkim indeksima raspodele RDW i PDW, značajno je povezana sa kliničkim ishodima kod pacijenata sa teškom traumatskom povredom mozga. Kombinovana procena biomarkera neuronalnog oštećenja i hematoloških parametara može predstavljati praktičan, laboratorijski zasnovan pristup ranoj prognostičkoj evaluaciji kod TTPM, iako je specifičnost kombinovanog modela u ovoj kohorti ostala ograničena.

## Introduction

Traumatic brain injury (TBI) represents a major global public health concern. It remains one of the leading causes of mortality and long-term neurological disability. It is estimated that approximately 5.48 million individuals worldwide suffer from craniocerebral injuries each year, among whom nearly one-fifth develop severe traumatic brain injury (STBI). STBI is characterised by a critical clinical condition, rapid disease progression, and poor prognosis, with reported mortality rates ranging from 30% to 50% [1]. Patients with STBI frequently present with impaired consciousness, inability to maintain adequate nutrition, and significant neurological dysfunction, which seriously affects functional recovery and quality of life [2]. Furthermore, secondary brain injuries, including subdural hematoma, epidural hematoma, intracerebral haemorrhage, and brain herniation caused by brainstem compression, may further aggravate neuronal damage and worsen patient outcomes [3]. Clinically, STBI is generally defined as a craniocerebral injury with a Glasgow Coma Scale (GCS) score ≤8 accompanied by altered mental status or loss of consciousness lasting more than 6 hours [4].

Early prognostic assessment plays an essential role in guiding treatment strategies and improving the clinical management of patients with STBI. The shock index (SI), defined as the ratio of heart rate to systolic blood pressure, is widely used for evaluating circulatory status and the severity of traumatic shock in emergency settings. Accurate assessment of SI upon admission can provide valuable information for early risk stratification and therapeutic decision-making [5]. In recent years, increasing attention has been paid to circulating biomarkers that reflect systemic inflammation, haematological alterations, and neuronal injury after traumatic brain injury.

Red cell distribution width (RDW) is a haematological parameter that reflects erythrocyte volume heterogeneity and is considered a potential marker of systemic inflammation and oxidative stress [6]. Elevated RDW levels have been associated with adverse outcomes in various critical illnesses. Platelet distribution width (PDW) reflects platelet size variability and may indicate platelet activation and inflammatory responses during disease progression [7]. In addition, neuron-specific enolase (NSE) is a glycolytic enzyme primarily located in neurons and neuroendocrine cells. Under physiological conditions, NSE concentrations in peripheral blood remain low; however, neuronal damage and disruption of the blood-brain barrier can lead to its release into the circulation, making it a useful biochemical marker of neuronal injury [8].

Given the potential of these circulating laboratory markers to reflect neuronal damage and systemic responses following trauma, their combined assessment may provide valuable prognostic information in STBI. Therefore, the present study aimed to evaluate the prognostic value of the reverse shock index multiplied by the Glasgow Coma Scale score (rSIG) in combination with RDW, PDW, and serum NSE levels in patients with severe traumatic brain injury, thereby providing laboratory-based evidence for improving early prognostic evaluation and clinical management of STBI patients.

## Materials and methods

### Study population

A retrospective analysis was conducted on the clinical data of 96 consecutive patients diagnosed with severe traumatic brain injury (STBI) who were admitted to our hospital between March 2020 and March 2023. Because the study was retrospective and included all eligible cases during the study period, no prospective sample size calculation was performed. According to the Glasgow Outcome Scale (GOS) evaluated 3 months after admission, patients were classified into a good-prognosis group (n = 46) and a poor-prognosis group (n = 50). Treatment strategies were individualised according to injury pattern and clinical status. They included conservative management, airway and ventilatory support, intracranial pressure control, hematoma evacuation, and decompressive craniectomy when indicated. The institutional Ethics Committee approved the study protocol, and all participants or their legal representatives provided written informed consent before inclusion in the study.

### Inclusion criteria

1. Glasgow Coma Scale (GCS) score ≤8 at admission;
2. admission within 12 hours after trauma;
3. diagnosis of severe craniocerebral injury confirmed by cranial imaging examinations such as computed tomography (CT) or magnetic resonance imaging (MRI);
4. age ≥18 years;
5. complete clinical and laboratory data available.

### Exclusion criteria

1. presence of other severe systemic injuries;
2. severe cardiac insufficiency, myocardial ischemia, or chronic renal failure;
3. central nervous system infections;
4. history of previous severe craniocerebral trauma;
5. anticoagulant disorders or anticoagulant therapy within six months before injury.

Patient prognosis was evaluated using the Glasgow Outcome Scale (GOS) [9]. A score of 1 indicates death, 2 indicates a vegetative state, 3 indicates severe disability, 4 indicates moderate disability with independent living, and 5 indicates good recovery with minor neurological deficits. GOS scores of 4-5 were defined as good prognosis, whereas scores of 1-3 were classified as poor prognosis. The 3-month follow-up point was selected because it was the most consistently available endpoint in the retrospective database and allowed assessment of early functional recovery after STBI; however, neurological recovery may continue beyond this period.

### Clinical and laboratory indicators

Baseline clinical data, including age, sex, heart rate, respiratory rate, and blood pressure, were recorded at admission. The reverse shock index multiplied by the Glasgow Coma Scale score (rSIG) was calculated to evaluate hemodynamic status [10]. The GCS value used for rSIG was the first documented admission score recorded in the emergency assessment before definitive neurosurgical intervention; in patients requiring early airway protection or sedation, the initial pre-intervention score in the chart was used whenever available.

In addition to clinical parameters, circulating laboratory biomarkers reflecting haematological alterations and neuronal injury were assessed. These included red cell distribution width (RDW), platelet distribution width (PDW), and serum neuron-specific enolase (NSE). RDW reflects erythrocyte size heterogeneity and is commonly used as an indicator of erythrocyte morphological variability and systemic inflammatory responses. PDW reflects platelet volume variation and may indicate platelet activation and inflammatory processes. NSE is a biochemical marker released from injured neurons and is widely used as a laboratory indicator of neuronal damage.

### Laboratory measurements

### Hematological analysis

Peripheral venous blood samples (2 mL) were collected from each patient under routine clinical conditions after hospital admission. Whole blood samples were analysed using an automated haematology analyser (Jinan Tai Doctor Physical Technology Co., Ltd., China) according to the manufacturer's instructions. RDW and PDW values were obtained as part of the complete blood count parameters generated by the analyser.

Quality control procedures were performed routinely before sample analysis to ensure the reliability and accuracy of haematological measurements. RDW was expressed as the percentage coefficient of variation of erythrocyte volume distribution, while PDW represented the variability in platelet size distribution derived from the platelet histogram generated by the haematology analyser.

### Biochemical detection of NSE

For biochemical analysis, 2 mL of fasting venous blood was collected in the morning. Blood samples were centrifuged at 1000 r/min for 10 minutes to obtain serum. The serum neuron-specific enolase (NSE) concentration was determined using enzyme-linked immunosorbent assay (ELISA) kits (Shanghai Fusheng Industrial Co., Ltd., China) according to the manufacturer's protocol. All samples were measured in accordance with standardised laboratory procedures to ensure analytical reliability.

### Calculation of rSIG

The reverse shock index multiplied by the Glasgow Coma Scale score (rSIG) was used as an indicator of physiological status in trauma patients. The rSIG value was calculated using the following formula: rSIG = GCS/SI, where SI (shock index) is defined as heart rate divided by systolic blood pressure [11][12]. Heart rate and systolic blood pressure were measured using an electronic sphygmomanometer (Shenzhen Taike Xinyuan Technology Co., Ltd., China).

### Statistical analysis

Statistical analyses were performed using SPSS version 20.0 (IBM Corp., Armonk, NY, USA). Continuous variables were expressed as mean ± standard deviation (x̄±s) and compared using an independent-samples t-test. Categorical variables were expressed as percentages and compared using the *x[* test. Because this was a retrospective exploratory study, no prospective sample size calculation was performed. Variables showing significant between-group differences in Table 1 were entered into the multivariable logistic regression model. Formal multicollinearity diagnostics were not available in the original dataset, and the regression results were therefore interpreted with caution. Receiver operating characteristic (ROC) curve analysis was used to evaluate the predictive performance of rSIG, RDW, PDW, and NSE for prognosis assessment. The area under the curve (AUC) was calculated to determine discriminative ability. A two-sided P value <0.05 was considered statistically significant.

**Table 1:** Comparison of clinical data of patients (x̄ ± s, %).

Clinical features	Good prognosis group<br>(n=46)	Poor prognosis group<br>(n=50)	t/c^2^	P
BMI (kg/m^2^)	23.92±2.01	23.19±2.18	1.701	0.092
Age (years)	56.42±5.83	57.06±5.42	0.557	0.579
Gender				
Male	30 (65.22)	33 (66.00)	0.007	0.936
Female	16 (34.78)	17 (34.00)		
Type of injury				
Simple extensive cerebral contusion	14 (30.43)	17 (34.00)	1.123	0.891
Extensive cerebral contusion<br>combined with epidural hematoma	6 (13.04)	8 (16.00)		
Combined subdural hematoma	8 (17.39)	9 (18.00)		
Combined intracerebral hematoma	9 (19.57)	6 (12.00)		
Combined multiple intracerebral<br>hematomas	9 (19.57)	10 (20.00)		
Heart rate (beats/min)	82.49±8.21	86.07±8.17	2.140	0.035
Respiratory rate (breaths/min)	20.03±2.12	21.15±2.28	2.486	0.015
Systolic blood pressure (mmHg)	127.04±8.27	122.19±10.18	2.549	0.012
Diastolic blood pressure (mmHg)	82.14±6.52	78.45±6.53	2.768	0.007
rSIG	10.65±1.14	6.74±1.26	15.895	0.000
RDW (%)	12.62±2.17	16.99±2.38	9.374	0.000
PDW (%)	15.31±2.05	19.64±2.12	10.156	0.000
NSE (ng/mL)	17.48±5.16	36.34±5.31	17.622	0.000

## Results

### Comparison of clinical and laboratory characteristics between prognosis groups

The baseline clinical and laboratory characteristics of the study population are summarised in Table 1. Compared with the poor-prognosis group, patients in the good-prognosis group exhibited significantly higher systolic and diastolic blood pressure at admission, as well as higher rSIG values (P<0.05). In contrast, the poor-prognosis group demonstrated significantly elevated heart and respiratory rates.

Notably, significant differences were also observed in circulating laboratory biomarkers. Patients with poor prognosis had markedly higher red cell distribution width (RDW), platelet distribution width (PDW), and serum neuron-specific enolase (NSE) levels than those with favourable outcomes (P<0.05). These findings suggest that increased haematological variability and elevated neuronal injury biomarkers are associated with unfavourable clinical outcomes in patients with severe traumatic brain injury.

### Logistic regression analysis of factors associated with poor prognosis in STBI

Variables showing statistically significant differences in Table 1 were further included in multivariable logistic regression analysis to identify factors independently associated with poor prognosis. In the regression model, outcome was coded as poor prognosis = 1 and good prognosis = 0. The analysis demonstrated that decreased rSIG values and elevated laboratory biomarker levels were independently associated with poor clinical outcomes. Specifically, lower rSIG (OR=0.224) and higher RDW (OR=3.010), PDW (OR=3.364), and NSE (OR=4.133) were isndependently associated with an unfavourable prognosis in patients with STBI (P<0.05). These results indicate that both physiological indices and circulating haematological and neuronal biomarkers contribute to prognostic evaluation. Detailed regression results are presented in Table 2.

**Table 2:** Results of multifactor logistic regression model analysis.

Risk factor	b	SE	Wald x^2^	OR	95% CI<br>upper limit	95% CI<br>lower limit	P
Heart rate (beats/min)	0.705	0.595	1.404	2.024	0.631	6.496	0.237
Respiratory rate (breaths/min)	0.527	0.403	1.710	1.694	0.769	3.732	0.192
Systolic blood pressure<br>(mmHg)	-0.785	0.682	1.325	0.456	0.120	1.736	0.250
Diastolic blood pressure<br>(mmHg)	-0.864	0.593	2.123	0.421	0.132	1.348	0.146
rSIG	-1.494	0.365	16.754	0.224	0.110	0.459	0.000
RDW (%)	1.102	0.398	7.666	3.010	1.380	6.567	0.006
PDW (%)	1.213	0.406	8.926	3.364	1.518	7.454	0.003
NSE (ng/mL)	1.419	0.457	9.641	4.133	1.688	10.122	0.002

### Predictive performance of rSIG and circulating biomarkers for the prognosis of STBI

**Figure 1:**
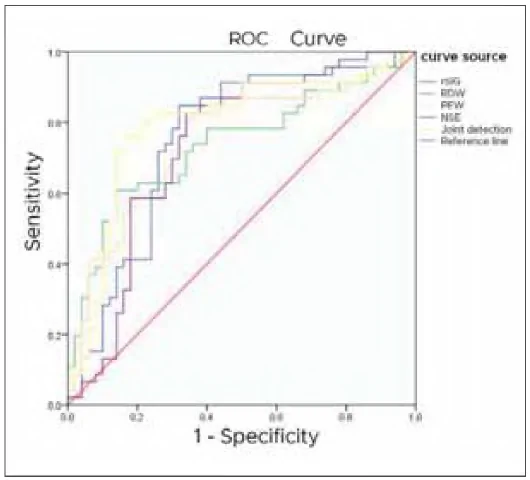
Graph of ROC result curve analysis.

Receiver operating characteristic (ROC) curve analysis was performed to evaluate the prognostic performance of rSIG and laboratory biomarkers for predicting poor outcomes in STBI patients. The results showed that rSIG demonstrated moderate predictive value with an area under the curve (AUC) of 0.752. Similarly, haematological and biochemical markers also showed predictive capability, with AUC values of 0.737 for RDW, 0.749 for PDW, and 0.736 for NSE. Importantly, the combined biomarker model integrating rSIG with RDW, PDW, and NSE achieved better overall discriminative performance, with an AUC of 0.793 (P<0.05). However, although sensitivity reached 95.00%, specificity was 50.00%, indicating that the combined model was more useful for screening or early risk identification than for highly specific classification. Detailed ROC analysis results are presented in Table 3, and the ROC curves are illustrated in Figure 1.

**Table 3:** Analysis of the predictive value of relevant indicators for the prognosis of STBI patients.

Indicator	AUC	95%CI	Sensitivity	Specificity	21.87 ng/mL	P
rSIG	0.752	0.650-0.853	84.78%	68.00%	9.286	0.000
RDW	0.737	0.635-0.839	60.87%	86.00%	16.25%	0.000
PDW	0.749	0.648-0.850	82.61%	64.00%	16.38%	0.000
NSE	0.736	0.632-0.839	82.61%	66.00%	21.87 ng/mL	0.000
Joint test	0.793	0.696-0.890	95.00%	50.00%	-	0.000

## Discussion

Severe traumatic brain injury (STBI) is frequently associated with elevated intracranial pressure, extensive neuronal damage, and a high risk of mortality and neurological disability [13]. As a major neurosurgical emergency, STBI is characterised by rapid clinical deterioration and poor outcomes in a substantial proportion of patients [14]. Although surgical interventions such as decompressive craniectomy can effectively relieve intracranial pressure and remove hematomas, the complexity of brain injury and secondary pathological processes often limits therapeutic success. Excessive decompression or severe tissue damage may further aggravate brain displacement and secondary injury, thereby increasing the risk of adverse neurological outcomes [15]. Therefore, identifying reliable laboratory biomarkers that reflect neuronal injury and systemic pathophysiological changes is essential for improving early prognostic assessment in STBI. At the same time, clinical outcomes after STBI may also be influenced by treatment strategy. Because management in this retrospective cohort was individualised and treatment types were not further stratified in the analysis, residual confounding related to treatment heterogeneity cannot be excluded.

Haematological indices obtained from routine blood tests have recently attracted increasing attention as potential biomarkers in critical illness. RDW reflects the heterogeneity of erythrocyte volume distribution and is considered an indicator of erythrocyte morphological variability. Elevated RDW levels have been associated with systemic inflammation, oxidative stress, and impaired erythropoiesis, and have been reported to predict adverse outcomes in various cardiovascular and critical diseases [16]. Similarly, PDW represents the variability in platelet size distribution and reflects platelet activation and turnover. Increased PDW may indicate enhanced platelet activation and inflammatory responses, both of which are important components of the pathophysiological cascade following traumatic brain injury [17].

In the present study, patients with poor prognosis exhibited significantly higher RDW, PDW, and NSE levels than those with favourable outcomes. These findings suggest that both haematological alterations and neuronal injury biomarkers are closely associated with clinical prognosis in STBI. The reverse shock index multiplied by the Glasgow Coma Scale score (rSIG) has been reported as a useful predictor of mortality in adult trauma patients [10]. to evaluate mortality risk in trauma patients. Previous studies have demonstrated that rSIG may provide better predictive performance for trauma severity than traditional shock indices, and higher rSIG values are generally associated with improved survival outcomes [10]. Our results are consistent with these findings, as patients in the good-prognosis group showed significantly higher rSIG values than those in the poor-outcome group.

The observed elevation of RDW and PDW in patients with an unfavourable prognosis may reflect systemic inflammatory responses and haematological dysregulation induced by traumatic injury. Increased RDW indicates greater erythrocyte size variability, which may result from impaired erythrocyte maturation, oxidative stress, inflammatory cytokine activity, or altered iron metabolism. These changes can reduce erythrocyte deformability and microcirculatory oxygen delivery, thereby potentially aggravating secondary cerebral ischemia after trauma. Similarly, elevated PDW suggests increased platelet activation and greater platelet heterogeneity. Activated platelets participate not only in hemostasis but also in endothelial injury, microthrombosis, leukocyte recruitment, and release of pro-inflammatory mediators, all of which may intensify secondary brain injury. In addition, NSE is a neuron-specific glycolytic enzyme primarily located in neuronal cytoplasm. Under physiological conditions, circulating NSE levels remain very low; however, disruption of neuronal membranes and damage to the blood-brain barrier can lead to its release into peripheral blood, making it a sensitive biochemical marker of neuronal injury. Therefore, elevated NSE may reflect both the extent of primary tissue destruction and the continuation of secondary injury processes [18].

Multivariable logistic regression analysis in this study demonstrated that decreased rSIG and increased levels of RDW, PDW, and NSE were independently associated with poor prognosis in patients with STBI. These findings suggest that both physiological status and circulating laboratory biomarkers contribute to outcome prediction. Previous studies have reported that elevated RDW is associated with unfavourable functional outcomes in ischemic stroke, platelet volume indices, including PDW, are associated with clinical outcome in acute ischemic stroke, and serum NSE has prognostic value in traumatic brain injury [16][17][18]. The present results further support the clinical relevance of these biomarkers for STBI prognosis assessment, although the limited sample size warrants cautious interpretation of the regression model's stability.

ROC curve analysis demonstrated that rSIG, RDW, PDW, and NSE each showed moderate predictive value for prognosis in STBI patients. Importantly, the combined biomarker model integrating clinical and laboratory parameters achieved improved overall discrimination, indicating that multi-parameter assessment may enhance early risk stratification. Nevertheless, the specificity of the combined model remained limited, so it should not be interpreted as a stand-alone diagnostic tool [19][20]. Several limitations should also be acknowledged. This was a single-centre retrospective study with a relatively small sample size, no prospective sample size calculation, and no formal multicollinearity diagnostics in the original analysis, all of which may have affected model stability. In addition, outcomes were assessed at 3 months, which captures early functional recovery but may underestimate later neurological improvement, and treatment heterogeneity was not analysed in detail. Larger prospective studies with longer follow-up and treatment-stratified validation are needed.

### Conclusions

In summary, decreased rSIG and elevated circulating levels of RDW, PDW, and NSE are significantly associated with poor clinical outcomes in patients with severe traumatic brain injury. These haematological and biochemical biomarkers reflect systemic inflammatory responses, platelet activation, and neuronal injury following traumatic brain damage. The combined assessment of rSIG with RDW, PDW, and NSE showed better overall discriminative performance than single indicators. Still, its specificity remained limited in this cohort. Therefore, integrated analysis of clinical indices and circulating laboratory biomarkers may provide a useful approach for early prognostic assessment and risk stratification in STBI patients, while external validation in larger cohorts is still required.

## Dodatak

### Conflict of interest statement

All the authors declare that they have no conflict of interest in this work.
